# Neuropsychiatric manifestations in juvenile systemic lupus erythematosus: what’s the weight of headache?

**DOI:** 10.1186/1546-0096-12-S1-P310

**Published:** 2014-09-17

**Authors:** Sofia Torreggiani, Maria Maddalena D'Errico, Giancarla Di Landro, Federica Cuoco, Antonella Petaccia, Alberto Cappellari, Giovanni Filocamo

**Affiliations:** 1 UOS Reumatologia Pediatrica, Fondazione IRCCS Ca’ Granda, Ospedale Maggiore Policlinico, Milano, Italy; 2 U.O.C. di Neurofisiopatologia, Dipartimento di Neuroscienze e di Salute Mentale, Fondazione IRCCS Ca' Granda Ospedale Maggiore Policlinico, Milano, Italy

## Introduction

Juvenile Systemic Lupus Erythematosus (JSLE) is an autoimmune disease characterized by multiple organ involvement. The nervous system is often affected, with a higher frequency in children than in adults.

## Objectives

To examine prevalence and features of neuropsychiatric manifestations in JSLE, focusing on headache.

## Methods

The records of all patients satisfying the ACR criteria for JSLE admitted to our Hospital from 1980 to May 2014 were retrospectively reviewed. Disease activity at onset was measured by SLE disease activity index (SLEDAI); organ damage was assessed at last follow-up using the SLICC/ACR Damage Index (SDI). Using the 1999 ACR nomenclature for neuropsychiatric (NP) lupus syndromes, patients were divided in patients with (NPSLE) and without (nNPSLE) NP involvement. Headache was defined as in the ACR nomenclature (ACR headache), as in SLEDAI (SLEDAI headache) and as “severe persistent headache; may be migrainous” (LHA).

## Results

Of 97 patients included in the study 84,5% were female. The female to male ratio was the same both in NPSLE and nNPSLE. Our population was predominantly white (90,7%). The mean ± SD age at onset was 11,8 ± 6,9 **y**ears and at diagnosis 12,9 ± 6,9 years. Mean ± SD length of follow-up was 6,6 ± 5,7 years. The mean ± SD age at onset of NP manifestations was 14,4 ± 9,3 years. The overall prevalence of NPSLE manifestations was 52,6%; NP syndromes were already present at the onset in 20,6% JSLE patients. In Table 1 we display the most common central nervous system syndromes reported in our NPSLE patients. The mean SLEDAI score at onset was 15,5 ± 7,7 in NPSLE and 12,6 ± 6,3 in nNPSLE. Mean SDI was 0,84 ± 0,9 in NPSLE and 0,5 ± 1,3 in nNPSLE. NP involvement was significantly associated with a SDI ≥ 1 (p < 0,05). Lupus anticoagulant (LAC) was present in 50,9% NPSLE and 19,6% nNPSLE patients. Antiphospholipid antibodies (APL) were detected in 50,9% NPSLE and 10% nNPSLE patients. In the subset of NPSLE patients, positivity of LAC and APL wasn’t significantly associated with headache. Magnetic Resonance Imaging (MRI) was performed in 37 NPSLE patients and was normal in 12. LHA definition was fulfilled in all patients with headache and pathological MRI.

**Table 1:** Most common central nervous system neuropsychiatric syndromes

	Overall (n. of patients)	At onset (n. of patients)	Later during the disease course (n. of patients)
Psychosis	5	2	3

Seizure disorders	7	5	2

Cerebrovascular disease	8	3	5

- ACR headache	37	9	28
- SLEDAI headache	1	-	1
- LHA	24	5	19

Movement disorder (chorea)	6	5	1

Mood disorder	8	2	6

## Conclusion

Consistently with the literature, more than a half of our JSLE patients presented NP involvement. Headache is a frequent manifestation: it was reported in 38,8% JSLE and 72,5% NPSLE patients. Headache in JSLE deserves an accurate investigation. In the subset of NPSLE patients, positivity of LAC and APL did not significantly correlate with headache, which may also be due to small number of cases. Further prospective studies are needed to better understand and define headache in JSLE.

## Disclosure of interest

None declared.

